# *Ulmus macrocarpa* Hance Extracts Attenuated H_2_O_2_ and UVB-Induced Skin Photo-Aging by Activating Antioxidant Enzymes and Inhibiting MAPK Pathways

**DOI:** 10.3390/ijms18061200

**Published:** 2017-06-05

**Authors:** Sun-Il Choi, Jin-Ha Lee, Jae-Min Kim, Tae-Dong Jung, Bong-Yeon Cho, Seung-Hyun Choi, Dae-Won Lee, Jinkyung Kim, Jong-Yea Kim, Ok-Hawn Lee

**Affiliations:** 1 Department of Food Science and Biotechnology, Kangwon National University, Chuncheon 24341, Korea; docgotack89@hanmail.net (S.-I.C.); tre98@hanmail.net (J.-H.L.); akxlwmf@nate.com (J.-M.K.); lgtjtd@naver.com (T.-D.J.); bongyeon.cho92@gmail.com (B.-Y.C.); zzaoszz@naver.com (S.-H.C.); jongkim@kangwon.ac.kr (J.-Y.K.); 2 Jeongseon Yaccho Co., Ltd., Jeongseon 26125, Korea; doll3261@naver.com (D.-W.L.); mintcandy802@hanmail.net (J.K.)

**Keywords:** *Ulmus macrocarpa* Hance extracts, human dermal fibroblasts, hairless mice, antioxidant, anti-aging

## Abstract

To protect from reactive oxygen species (ROS) damages, skin cells have evolved to have antioxidant enzymes, such as copper and zinc-dependent superoxide dismutase (SOD1), mitochondrial manganese-dependent superoxide dismutase (SOD2), catalase (CAT), glutathione peroxidase (GPX), and glutathione reductase (GR), and suppressed the expression of matrix metalloproteinases (MMPs) through the mitogen-activated protein kinase (MAPK) signaling pathways, such as c-Jun N-terminal kinase (JNK) and p38. Bioactive compounds analyses were performed using a high-performance liquid chromatography-photodiode array detector (HPLC-PDA) system. The antioxidant activity of *Ulmus macrocarpa* Hance (UMH) extracts was estimated in vitro. The anti-aging activity of UMH extracts was estimated in vivo using the SKH-1 hairless mice. The UMH extracts reduced the H_2_O_2_-induced intracellular ROS production and the cell damages in human dermal fibroblasts (HDFs). Moreover, the H_2_O_2_-induced phosphorylation of JNK and p38 was detected in HDF and UMH extracts blocked the phosphorylation. These results suggest that UMH extracts can reduce the expression of MMPs and the reduced MMPs lead to the inhibition of collagen degradation. In addition, oral administration of the UMH extracts decreased the depth, thickness, and length of wrinkles on UVB exposed hairless mice. Therefore, UMH extracts play an advantage of the functional materials in antioxidant and anti-aging of skin.

## 1. Introduction

Skin surrounding the human body is exposed to the outside and protects the body from external stimulation. Skin aging can be classified as intrinsic aging and photo-aging. The first proceeds with time passing away naturally and the latter occurs in the skin of the face, back of the hand, or neck after overexposure to sunlight. In general, ultraviolet (UV) is the ultimate reason for photo-aging in the skin and intrinsic aging is the result of combinatorial factors [1]. UV light, which is a cause of photo-aging, can be classified into UVA (320–400 nm), UVB (280–320 nm), and UVC (200–280 nm). UVA and UVB from the Earth’s surface pass through the dermal layer and promote damage and aging in skin [2]. Light absorbed into the skin transmits its energy to various plastids, such as nucleic acids, amino acids, and melanin. Next, the plastid generates free radicals, causing photolysis and creates a superoxide anion radical by interacting with free oxygen molecules present in the living body. These radicals are subjected to chain reactions to create a harmful ROS, such as singlet oxygen (^1^O_2_), hydroxyl radical (⦁OH), and hydrogen peroxide (H_2_O_2_) [3].

Reactive oxygen species (ROS) lead the cell and tissue damage in skin. In particular, ROS destroy antioxidant enzymes such as glutathione reductase (GR), superoxide dismutase (SOD), glutathione peroxidase (GPX), and catalase (CAT) [4]. Additionally, the overproduction of ROS can result in apoptosis or necrosis. The most significant effects of these processes are found in the mitogen-activated protein kinase (MAPK)/activator protein-1 (AP-1) [5]. Therefore, the way of protecting the skin from UV and preventing aging inhibits the oxidative stress caused by ROS. Thus, the study of natural resource with antioxidant to remove ROS is being actively pursued [6].

*Ulmus macrocarpa* Hance selected in this study is that the dried bark of the *Ulmus davidiana* var. *japonica* N. *Ulmus macrocarpa* Hance has been used as an oriental medicine for decades in the treatment of severe metabolic diseases, such as inflammation, edema, gastric cancer, and anti-bacterial infections in South Korea [7]. However, the exact mode of its action to metabolic diseases has never been elucidated.

In this study, we investigated the antioxidant and anti-aging effects of *Ulmus macrocarpa* Hance (UMH) extracts on the H_2_O_2_-induced ROS in human dermal fibroblasts (HDFs) and UVB-exposed hairless mice.

## 2. Results

### 2.1. Various Antioxidant Compounds in Ulmus macrocarpa Hance (UMH) Extracts

Plants contain many phytochemicals such as phenolics, which have antioxidative effects. Thus, we determined the various antioxidant compounds of UMH extracts. It had the mean value of antioxidant compounds content as catechin of 7.84 ± 0.04 mg/g. In addition, total phenolic content and total proanthocyanidin contents were assessed as 404.25 ± 2.48 mg GAE/g and 239.82 ± 8.45 mg CE/g, respectively.

### 2.2. Antioxidant Activity of UMH Extracts

**Figure 1:**
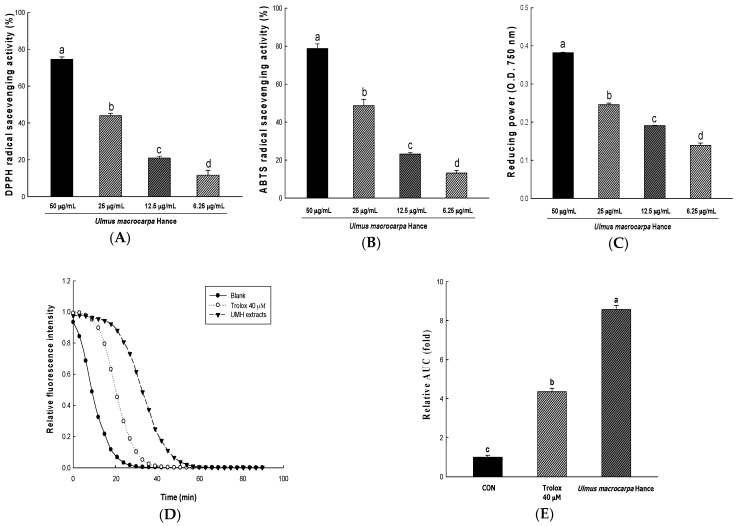
Antioxidant activities of *Ulmus macrocarpa* Hance (UMH) extract. 2,2-Diphenyl-1-picrylhydrazyl (DPPH) radical scavenging activity (**A**); ABTS radical scavenging activity (**B**); reducing power (**C**) of *Ulmus macrocarpa* Hance at various concentrations. Radical scavenging activity of UMH extracts were measured by oxygen radical antioxidant capacity (ORAC). Decay curve of fluorescence in black, trolox 40 μM and UMH extracts (**D**,**E**). ^a^^–^^d^ All values are presented as the means ± standard deviation (SD). The groups of different concentrations of UMH were compared with each other and letters on the top of the columns do not share the same letters are statistically significant differences among the experimental groups at *p* < 0.05 by one-way ANOVA.

In order to measure the anti-oxidant activity of UMH extracts, we used in vitro methods, such as reducing power, 2,2-diphenyl-1-picrylhydrazyl (DPPH) and 2,2′-azino-bis(3-ethylbenzothiazoline-6-sulfonic acid) diammonium salt (ABTS) radical scavenging activities. The foregoing method measures the anti-oxidant levels through different mechanisms. The DPPH radical scavenging activities of UMH extracts in 6.25, 12.5, 25 and 50 μg/mL are each 11.65 ± 2.69%, 21.00 ± 0.95%, 43.95 ± 1.27%, and 74.53 ± 1.30%, respectively (IC_50_: 31.65 ± 0.13 μg/mL). The DPPH radical scavenging activities were increased depending on the concentration of UMH extracts (Figure 1A). ABTS radical scavenging is a mechanism similar to DPPH radical scavenging. The values were 13.14 ± 1.31%, 23.16 ± 0.90%, 48.66 ± 3.34%, and 78.75 ± 2.49% in 6.25, 12.5, 25, and 50 μg/mL of UMH extracts, respectively (IC_50_: 25.95 ± 1.59 μg/mL). ABTS radical scavenging also increased in a dose-dependent manner (Figure 1B). Reducing power at the anti-oxidant level acts through a different mechanism and is a method to measure the degree of reduction from oxidation. The capacities increased in a dose-dependent manner (Figure 1C). The antioxidant capacity of UMH extracts for the oxygen radical absorbance capacity (ORAC) assay is shown in Figure 1D, Figure 1E showed the AUC of the UMH compared with blank AUC. In addition, ORAC values are expressed as Trolox equivalents (μmol) by gram of extracts (3426.49 ± 69.13 μM TE/g).

### 2.3. UMH Extracts Protect Human Dermal Fibroblasts (HDFs) from H_2_O_2_-Induced Cell Death

**Figure 2:**
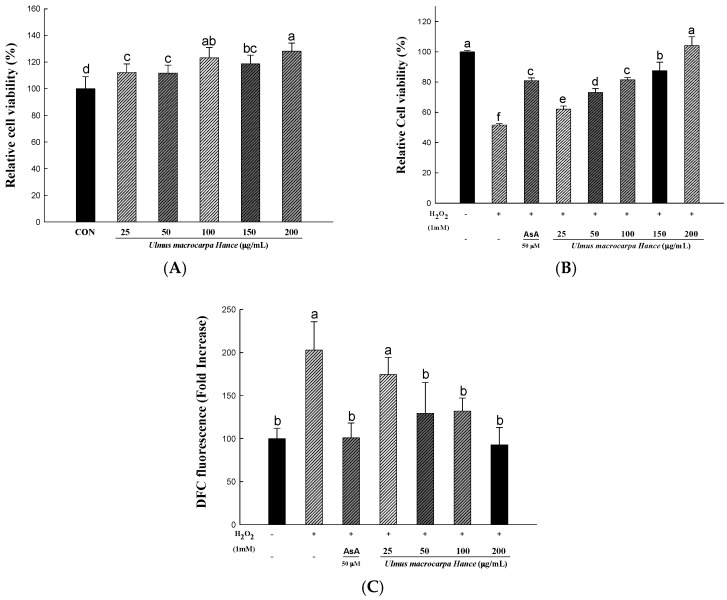
Cell viability and intracellular reactive oxygen species (ROS) generation in human dermal fibroblasts (HDFs) treated with UMH. (**A**) Cell viability of HDFs treated with or without UMH extracts; (**B**) The protective effect of UMH extracts on H_2_O_2_-treated HDFs; (**C**) the intracellular ROS levels by fluorescence. Intracellular ROS generation was measured by cell-permeant 2′,7′-dichlorodihydrofluorescein diacetate (H_2_DCFDA) assay. The cell was exposed to UMH for 24 h, then 1 mM H_2_O_2_ for 2 h. ^a–f^ All values are presented as means ± SD. The groups of control, H_2_O_2_, ascorbic acid (AsA), and UMH were compared with each other and different letters on the top of the columns do not share the same letters are statistically significant differences among experimental groups at *p* < 0.05 by one-way ANOVA.

In order to determine the cytotoxicity and concentration ranges of UMH extracts, we used an 2,3-*bis*-(2-methoxy-4-nitro-5-sulfophenyl)-2H-tetrazolium-5-carboxanilide (XTT) assay. For treating the UMH extracts, the cytotoxicity of the sample extract was analyzed by calculating the degree of change in cell growth as a percentage, compared to the mock control group. As shown in Figure 2A, the cytotoxicity was not shown in all samples. Additionally, Figure 2B shows the results of the cell protective effect from H_2_O_2_-induced cell damage. It decreased to 51% compared to the control group, without any processing, only when treating H_2_O_2_ in HDFs. Additionally, the ascorbic acid used as a positive control to protect from cell damage and recover from 51% caused by H_2_O_2_ to 81%. In the case of UMH extracts, the concentration of 100 μg/mL showed a similar level as the effect of ascorbic acid and the 200 μg/mL showed a similar level as the effect of the control group without any processing. In addition, the cell viability by UMH extracts was increased in a dose-dependent manner. Cells treated with H_2_O_2_ exhibited the cell damage-like morphological characteristics, such as detachment and cytoplasmic shrinkage [8]. These results confirmed that UMH extracts had an adequate protective effect against H_2_O_2_-mediated cell damage. Similarly, H_2_O_2_ was reported by Park et al. [9] to induce oxidative stress in HDFs, reduce cell viability, and increase the rate of morphological cell damage.

### 2.4. UMH Extracts Inhibit H_2_O_2_- to Induce ROS Production in HDFs

The cell-permeant 2′,7′-dichlorodihydrofluorescein diacetate (H_2_DCFDA) assay is widely used for directly measuring the reductive and oxidative states of cells. The method to changes in the redox state of cells and can be used to measure changes in ROS levels over time [10]. To study the intracellular ROS scavenging activity of UMH extracts, the ROS-sensitive fluorescent probe, H_2_DCFDA, was used. The H_2_O_2_ treatment induces the intracellular ROS. As shown in Figure 2C, pretreatment with UMH extracts or ascorbic acid for 24 h, H_2_O_2_ treatment induced a 2′,7′-dichlorofluorescein (DCF) fluorescence intensity 1.8-fold higher than the control group. As a result, the pretreatment with UMH extracts for 24 h decreased the fluorescence intensity in a dose-dependent manner.

### 2.5. UMH Extracts Inhibit Premature Senescence Induced by Hydrogen Peroxide in HDFs

**Figure 3:**
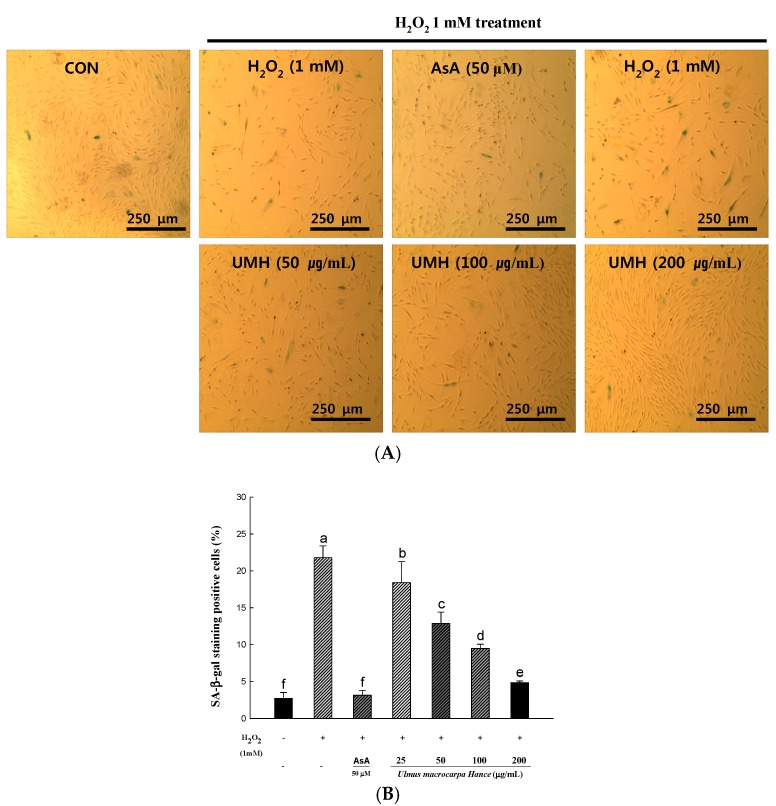
The suppressible effect of UMH on H_2_O_2_-induced expression of senescence-associated β-galactosidase (SA-β-gal) in HDFs. The photographic images (**A**) and the representative percentage of X-gal positive cells (**B**). ^a–f^ All values are presented as means ± SD. The groups of control, H_2_O_2_, AsA and UMH were compared with each other and letters on the top of the columns do not share the same letters are statistically significant differences among experimental groups at *p* < 0.05 by one-way ANOVA.

Oxidative stress has been shown to induce stress-induced premature senescence in fibroblasts [11]. In response to replication and serial passage, senescence-associated β-galactosidase (SA-β-gal) positive staining is increased. SA-β-gal activity is used commonly as a senescent marker. To confirm that UMH extracts play a role in H_2_O_2_-induced premature senescence, we measured the SA-β-gal activity and monitored the morphological appearance of the enlarged and flattened cells. After treatment with H_2_O_2_, the percentage (21%) of senescent cells increased significantly (Figure 3B). However, pretreatment with UMH extracts for 24 h decreased the SA-β-gal activity in a dose-dependent manner. Photographs representing cells with SA-β-gal positive staining are shown in Figure 3A. The cells showed significant enlargement and flattening in H_2_O_2_-induced premature senescence. 

### 2.6. UMH on the Expression of Cellular Antioxidant Enzymes

**Figure 4:**
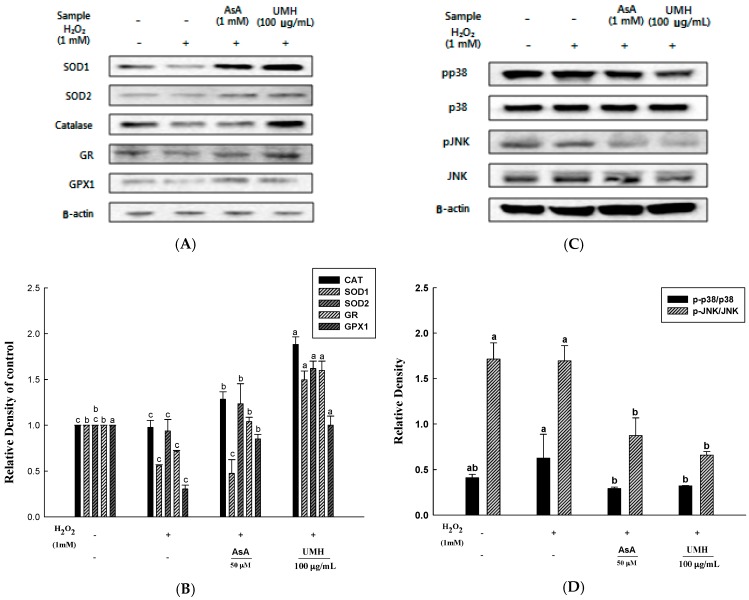
Western blot analyses of antioxidant enzyme expression inhibitory effects of UMH on the MAPK pathway. Confluent cells were incubated with bark extracts of UMH for 24 h prior to exposure to 1 mM H_2_O_2_ for 3 h. (**A**) The expression levels of the zinc-dependent superoxide dismutase (SOD1), mitochondrial manganese-dependent superoxide dismutase (SOD2), catalase (CAT), glutathione reductase (GR), and glutathione peroxidase (GPX1) proteins were determined by Western blot, respectively; (**B**) Protein levels were normalized by β-actin immune content and expressed as a percentage of control; (**C**) The expression levels of the p38, pp38, JNK, and pJNK proteins were determined by Western blot, respectively; (**D**) The ratio of pp38/p38 and pJNK/JNK. ^a–c^ All values are presented as means ± SD. The control, H_2_O_2_, AsA, and UMH groups were compared in the same color of column, and letters on the top of the columns do not share the same letters are statistically significant differences among experimental groups at *p* < 0.05 by one-way ANOVA.

The induction of the activity in a particular enzyme group is considered to play an important role in the cellular defense strategy against oxidative stress, caused by high concentration of toxic metal [12]. We aimed to ascertain the existence of a causal relationship between the improved expressions of antioxidant enzymes and H_2_O_2_-treated cells controlled with UMH extracts. Among antioxidant enzymes, we checked zinc-dependent superoxide dismutase (SOD1), mitochondrial manganese-dependent superoxide dismutase (SOD2), catalase (CAT), glutathione reductase (GR), and glutathione peroxidase (GPX1) (Figure 4A). The levels of SOD1 and SOD2 proteins were increased significantly upon the addition of ascorbic acid and UMH extracts in an H_2_O_2_-induced oxidative stress model. Additionally, the level of CAT protein was increased significantly upon UMH extracts. Furthermore, the increase in levels of GR and GPX1 proteins were critical upon ascorbic acid and UMH extracts, especially UMH extracts. Therefore, these expressions of antioxidant enzymes are significant that UMH extracts inhibited H_2_O_2_-induced ROS.

### 2.7. UMH Blockade of c-Jun N-Terminal Kinase (JNK) and p38 MAPK Signaling Pathways

The pathway of MAPK signal transduction plays an important role in regulating a variety of cellular functions, including matrix metalloproteinases (MMPs) expression [13]. MMP-1 is by decomposing collagen, leading to the reduced elasticity and wrinkle formation of the connective tissues.

The effect of UMH extracts on MAPK signaling pathways was analyzed by Western blot (Figure 4C). The H_2_O_2_ treated group revealed that the levels of phosphate-JNK (c-Jun N-terminal kinase) (1.1-fold of JNK) and phosphate-p38 (1.2-fold of p38) proteins were significantly higher than a control. However, ascorbic acid which is a positive control and UMH extracts treated groups effectively suppressed the activation of phosphate-JNK (0.6- and 0.4-fold of JNK, respectively), as well as phosphate-p38 (0.8- and 0.5-fold of p38, respectively) induced by H_2_O_2_. These results support that the expression of MMP by JNK and the p38 signaling pathway.

### 2.8. UMH Inhibit UVB-Induced Photo-Aging in Skin of Hairless Mice

**Figure 5:**
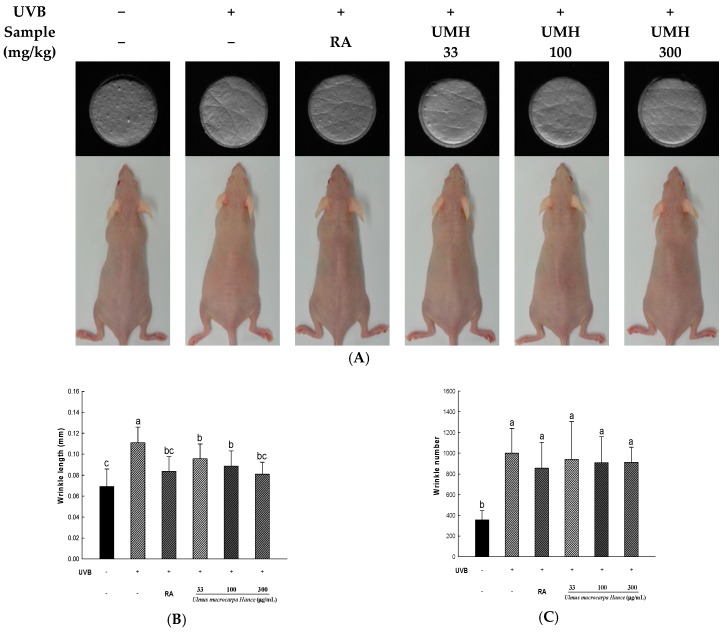

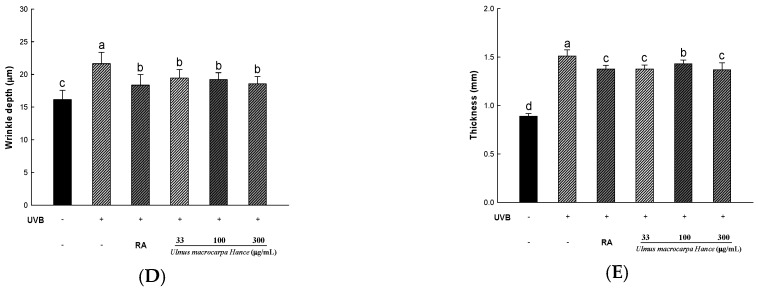
The effects of UMH extracts in hairless mice on UVB-induced wrinkle formation. (**A**) Characteristics of the dorsal skin of hairless mice exposed UVB for 10 weeks. (**B–E**) Skin impressions were analysed after 10 weeks of treatment. ^a–c^ All values are presented as means ± SD. The groups of control, UVB, RA and UMH were compared with each other and letters on the top of the columns do not share the same letters are statistically significant differences among experimental groups at *p* < 0.05 by one-way ANOVA.

In this study, using a photo-aging caused by UVB-induced hairless mice, we examined inhibitory effect for wrinkle formation of a well-known antioxidant as retinoic acid and UMH extracts. After five weeks of applying UVB to hairless mice, wrinkle formation in hairless mice was detected by the naked eye. After 10 weeks of irradiation with UVB, the changes in the skin folds were compared to the control (Figure 5). The thickness, length, and depth of the formed wrinkles in the UVB-irradiated group were compared to those of the UVB-non-irradiated group. However, in a positive control in which 0.01% retinoic acid was applied and the group of orally-administered UMH extracts, wrinkle formation detected by the naked eye was less than the experimental group. Especially, the depth, thickness, and length of wrinkles confirmed the inhibitory effect in the higher concentration of UMH extracts.

## 3. Discussion

Skin is exposed to the outside and serves to protect the body from external stimulation. In addition, the skin is the external beauty and health to the modern people’s pursuit of quality of life. Therefore, healthy skin is gaining attention as an important aspect of age reversing. Aging due to sunlight is called photo-aging. In particular, ultraviolet rays such as UVB are known to induce oxidative stress, which cause ROS to form in the skin and tissue and to reduce antioxidant production [14]. In addition, the ROS generated in this process are known to produce wrinkles, reducing the synthesis of collagen fibers and elastic fibers. ROS has also been reported to cause the body to oxidize components such as protein, fat, and cell membranes, promoting skin aging [15]. Therefore, the study of natural substances with antioxidant activity which removes ROS and suppresses the oxidative stress caused by free radicals is progressing to protect the skin from the UVB and to prevent aging [6]. Our study showed the antioxidant and anti-aging effects on ROS production of UMH extracts contain a lot of phytochemicals such as phenolic compounds. Phenolic compounds have been reported to have antioxidant, anti-aging, anti-tumor, and inhibition of hyperlipidemia as a mechanism for receiving the electrons within the phenolic hydroxyl groups in molecule [16,17]. In this study, UMH extract is confirmed to contain high levels of phenolic compounds and antioxidant activity. Antioxidant activity was measured using DPPH radical scavenging activity, ABTS radical scavenging activity, and the reducing power of UMH at various concentrations. 

DPPH radicals interrupt the formation of a stable molecule. It accepts an electron or hydrogen radical by ascorbic acid, tocopherol, polyhydroxy aromatic compounds, and aromatic amine, and triggers a color change at different concentrations [18]. Additionally, ABTS radical scavenging activity was used in the generated ABTS⦁^+^ by reaction with potassium persulfate. The ABTS⦁^+^ reacts with the anti-oxidizing material in a sample and changes the colors at different concentrations at 734 nm [19]. Another measurement of anti-oxidant activity, reducing power, is associated with the presence of reductones known to exert anti-oxidant activity by destroying the free radical chain through the donation of a hydrogen atom [20]. Ferric-ferricyanide (Fe^3+^) stabilizes the free radical by sharing a hydrogen atom. Then, ferric-ferricyanide (Fe^3+^) is reduced to ferrous (Fe^2+^) and the reducing power represents the absorbance value (700 nm) [21]. Lastly, the ORAC assay is a method of measuring the anti-oxidant activity of the sample using the reduction of fluorescence by peroxyl radicals. When the peroxyl radical is generated by 2,2′-azobis (2-methylpropionamidine) dihychloride, the antioxidants of the sample maintain the fluorescence by removing the peroxyl radical and are detected to prevent the oxidation of the peroxyl radical until the anti-oxidants of the sample are depleted. The antioxidant capacity of the sample across time is represented by the decay curve and quantified by comparison to the standard, which is water soluble vitamin E (Trolox) [22]. Kim et al. [23] reported that the *Ulmus pumila* L. which is the peel of roots of the *Ulmus davidiana* var. *japonica* N. contains superior antioxidant activity. In our study, *Ulmus macrocarpa* Hance, which is the stem bark of the *Ulmus davidiana* var. *japonica* N., has been confirmed the effectiveness of the antioxidant levels similar to *Ulmus pumila* L. Additionally, we propose that UMH extracts inhibit of H_2_O_2_-induced ROS damage by facilitating the expression of antioxidant enzymes, such as CAT, GR, SOD1, SOD2, and GPX1. Our result show the increased expression of these enzymes with UMH extracts, suggesting that the UMH inhibited H_2_O_2_-induced ROS.

UVB irradiation and ROS can induce MMP expression by activating transcription factors through the MAPK signaling pathway, such as JNK and p38 [24]. Our results showed that, H_2_O_2_-induced phosphorylation of JNK and p38 was detected and UMH extracts blocked the phosphorylation of JNK and p38. These results suggest that the increased expression of MMPs lead to collagen degradation, which leading to wrinkle formation [25]. Furthermore, wrinkle formation in skin was examined in SKH-1 hairless mice exposed to UVB radiation for 10 weeks. As a result, UMH extracts significantly alleviated the levels of thickness, length, and depth of the formed wrinkles.

**Figure 6:**
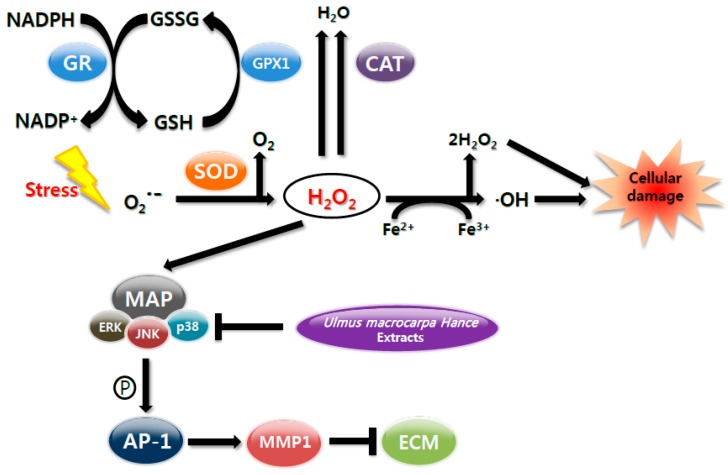
The proposed model of UMH extracts’ inhibitory action in ROS-induced cellular senescence. These mechanisms indicated that UMH extracts had an adequate antioxidant and anti-aging effects on H_2_O_2_-induced reactive oxygen species (ROS) generation. ROS cause a reduction of superoxide dismutase (SOD), catalase (CAT), glutathione peroxidase (GPX), and glutathione reductase (GR), followed by activation of c-Jun N-terminal kinase (JNK), p38 mitogen-activated protein kinase (p38), and matrix metalloproteinase (MMPs) activation.

In conclusion, the present results demonstrate that UMH extracts contain many phenolic compounds and high levels of antioxidant activity. UMH extracts enhanced the expression of antioxidant enzymes such as SOD1, SOD2, CAT, GPX1, and GR in response to H_2_O_2_-induced oxidative stress in HDFs. Additionally, UMH extracts are a potent inhibitor of the H_2_O_2_-induced block of the MAPK signaling pathway in HDFs. Furthermore, it inhibited the UVB-induced wrinkle formation in skin of UVB-exposed hairless mice. Taken together, UMH extracts are considered as advantageous functional materials to prevent oxidative stress-induced premature skin aging (Figure 6).

## 4. Materials and Methods

### 4.1. Materials

Dried samples of UMH were extracted with 30% ethanol at 60 °C for 3 h. The extract was filtered (No.3, Whatman, Kent, UK) and concentrated with a rotary evaporator (N-3000, Eyela, Tokyo, Japan). The extracted materials were freeze-dried (Biotron, Bucheon, Korea). The extracts were dissolved in the dimethyl sulfoxide (DMSO) and used in the experiment. Trypsin- ethylenediaminetetraacetic acid (EDTA), fetal bovine serum (FBS), Dulbecco’s modified Eagle’s medium (DMEM), and phosphate buffered saline (PBS) were obtained from Gibco (Gaithersburg, MD, USA). 2,2′-azino-*bis*(3-ethylbenzothiazoline-6-sulfonic acid) diammonium salt (ABTS), 1,1-diphenyl-2-picryl hydrazyl radical (DPPH), sodium carbonate, 2,4,6-tris(2-pyridyl)-s-triazine (TPTZ), 6-hydroxy-2,5,7,8,-tetramethylchroman-2-carboxylic acid (Trolox), sodium phosphate dibasic, potassium ferrocyanide, 2,2′-azobis(2-methylpropionamidine) dihydrochloride (AAPH), Folin-Ciocalteu’s phenol reagent, potassium persulfate, sodium carbonate, penicillin–streptomycin (P/S) hydrogen peroxide (H_2_O_2_), and Triton X-100 were purchased from Sigma-Aldrich Co. (Saint Louis, MO, USA).

### 4.2. Determination of the Phenol Compounds

Analysis of catechin was done using a HPLC/PDA system (NANOSPACE SI-2 series, Shiseido Co., Ltd., Tokyo, Japan). Separation was performed on a SunFire™ C_18_ column (250 mm × 4.6 mm, 5 μm particle size) (Shiseido Co., Ltd.). A sample volume of 10 μL was injected into the column and eluted with a constant flow rate of 1.0 mL/min. The mobile phase was consisted of 60% acetonitrile with 50 mM KH_2_PO_4_. Continuous scanning was performed by UV detector, and chromatograms were acquired at 254 nm for 15 min.

To determine of the total phenolic contents in UMH extracts, used by the method of Gutfinger [26] with some modifications. The sample solution (500 μL) were added with (12.5%, *w*/*v*) sodium carbonate solution (1.25 mL) and incubated at 25 °C for 40 min after which 1.0 M Folin-Ciocalteu reagent (250 μL) was added. The absorbance was measured at 750 nm and, gallic acid was used as the calibration curve.

To determine of the total proanthocyanidin contents in UMH extracts, using the method of Mitsunaga et al. [27] with some modifications. The sample (0.5 mg) was diluted with 5 mL methanol. The sample solution was placed in a tube with 3 mL vanillin (4%, *w*/*v*) and 1.5 mL of concentrated hydrochloric acid and incubated for 15 min at room temperature. The absorbance was determined at 429 nm, and catechin was used as the calibration curve.

### 4.3. Determination of the Antioxidant Activity

To determine of the antioxidant activity such as DPPH and ABTS radical scavenging activity, reducing power and Oxygen radical absorbance capacity, described previously study of Lee et al. [28]. For DPPH radical scavenging activity, the method of Chu et al. [29] was used with some modifications. The various concentrations of sample solution (0.2 mL) or control were allowed to react with DPPH solution (0.8 mL) for 30 min in the dark at room temperature. Then the absorbance was taken at 515 nm and the radical scavenging activity was calculated and expressed as a percentage using the following Formula (1).

In the case of ABTS radical scavenging activity, the method of Robert et al. [30] was used with some modifications. The various concentrations sample solution (50 μL) or control were allowed to react with ABTS⦁^+^ solution (150 μL) for 20 min. Then the absorbance was taken at 734 nm and the radical scavenging activity was calculated and expressed as a percentage using the following formula:

Radical scavenging activity (%) = [(Ac − As)/Ac] × 100(1)

where Ac is the absorbance of the control and As is the absorbance of the sample.

The reducing power assay, the method of Oyaizu [21] was used with some alterations. The various concentrations of sample solutions were allowed to mix with 0.5 mL of sodium phosphate buffer (0.2 M) and 0.5 mL of potassium ferricyanide (1%, *v*/*v*) for 20 min at 50 °C. After incubating, 0.5 mL of trichloroacetic acid (10%, *w*/*v*) was added and the mixture was centrifuged at 1790× *g* for 10 min. A 2.5-mL aliquot of the supernatant was mixed with 2.5 mL of distilled water and 0.2 mL of Iron(Ш) chloride (0.1%, *w*/*v*). Then the absorbance was taken at 700 nm.

The ORAC assay, the method of Ou et al. [22] was used with some modifications. The various concentrations of samples were dissolved in 75 mM phosphate buffer (pH 7.4) at 37 °C. The sample solutions (25 μL) were allowed to incubating with 40 nM fluorescein (150 μL) and 18 mM AAPH (25 μL) into black 96-well plates for 15 min at 37 °C. Then, to estimate the fluorescence, the plate was immediately transferred to a fluorescence microplate reader (Spectramax GEMINI EM, Molecular Devices, Sunnyvale, CA, USA). The reader record the fluorescence of FL every 3 min for 90 min at emission and excitation wavelengths of 520 and 485 nm, respectively. Additionalyl, Trolox was used as a standard for the calibration curve.

### 4.4. Cell Culture and Cell Viability

HDFs were provided from the American Type Culture Collection (ATCC). Briefly, cells were plated at 37 °C in a 5% CO_2_ atmosphere and grown in DMEM with 10% FBS media. Then, we replaced with a new medium every 2–3 days. HDFs were treated 24 h before treatment with H_2_O_2_ for 1.5 h to simulate oxidative damage.

Cell proliferation was detected by the XTT assay (WelGene, Seoul, Korea). When the cells were cultured to the log phase, the cells were seeded on a 96-well plate (1 × 10^5^ cells/well). After 24 h, treatment UMH extracts concentration groups for 24 h. To measure and eliminate, absorbance values were measured at 450 nm for XTT and 690 for background.

Cell viability (%) = (As/Ac) × 100%(2)

where Ac is the absorbance of the control and As is the absorbance of the sample.

### 4.5. Measurement of Intracellular Reactive Oxygen Species (ROS) Generation

Intracellular ROS generation was determined by H_2_DCFDA assay. Briefly, cultured cells were fixed with 4% paraformaldehyde for 15min at room temperature. Fixed cells were incubated for 150 min at 37 °C with a 10 μM H_2_DCFDA solution in PBS labelled with fluorescein intracellular H_2_O_2_. Intracellular ROS generation was observed by fluorescent microscopy (Leica DMI 3000B, Wetzlar, Germany). To quantitatively measure ROS levels, relative fluorescence was determined by a fluorescence microplate reader at excitation and emission wavelengths of 485 and 535 nm, respectively.

### 4.6. Senescence-Associated β-Galactosidase (SA-β-Gal) Staining

SA-β-gal can be used to specifically identify senescent cells. The cultured cells were washed with PBS and fixed with 4% paraformaldehyde for 15 min at room temperature. The fixed cells were stained with X-gal solution (5 mM potassium ferricyanide, 5 mM potassium ferrocyanide, 1 mg/mL of 5-bromo-4-chloro-3-indolyl β-d-galactoside and PBS).The cells were incubated at 37 °C for 48 h washed with PBS. Senescent cells were identified as blue-stained cells by standard optical microscopy (Olympus, Tokyo, Japan) and five arbitrary fields were used to determine the proportion of positive staining cells.

### 4.7. Western Blot Analysis

The HDFs were washed twice, harvested in PBS and carried out using whole cell lysates prepared. Cell lysates were obtained from equal amount of total protein, or the equal volume of supernatant was electrophoresed on 10% sodium dodecyl sulfate polyacrylamide gel electrophoresis (SDS-PAGE) gels and the polyvinylidene difluoride membrane (Bio-Rad, Hercules, CA, USA). Nonspecific binding was blocked using TBS-T buffer containing 5% skin milk for 1 h and the membranes were incubated with primary antibodies at 4 °C overnight. Finally, the membranes were treated with secondary antibodies for 1 h at room temperature and washed with TBS-T. Each protein was detected by Super Signal West Pico Chemiluminescence detection reagents (Pierce Biotechnology, Rockford, IL, USA) and a digital camera using the Chemi Doc image software program (Bio-Rad).

### 4.8. UV Irradiation of Hairless Mice

Six-week-old male SKH-1 hairless mice were purchased from Daehan Biolink Ltd. (Ochang, Korea). Mice were housed in a climate control room (22 °C at 50% humidity) with a 12 h light/dark cycles and were free to consume water and food. The trial was approved by the Institutional Animal Care and Use Committee of Chungbuk Technopark—IACUC number EBOA-2015-10 (7 Augest 2015). Written informed consent was obtained from all participants. The mice were assigned to six groups, including the normal (*n* = 6), the UVB-irradiated vehicle (*n* = 6), the UVB-irradiated retinoic acid (*n* = 6), the UVB-irradiated with 33 mg/kg (*n* = 6), 100 mg/kg (*n* = 6) and 300 mg/kg (*n* = 6) groups. Groups of UVB-irradiated mice taking various concentrations of UMH extracts were orally administered according to body weight per day. Drinking water was supplied to all of the groups.

The minimal erythema dose (MED) on the dorsal skin of the mice was investigated. UVB light (Phillips, Eindhoven, The Netherlands) that emitted UVB in the range of 280 to 360 nm was used as the source to irradiate the dorsal skin of mice. The backs of mice were exposed to UVB three times a week. During the first week, a UVB dose of 1 MED (150 mJ/cm^2^) was applied. The intensity was then increased by 1 MED per week for up to five weeks, after which the mice were exposed to 5 MED for 10 weeks so that UV irradiation during the test period amounted to 90 MED (13,500 mJ/cm^2^).

### 4.9. Wrinkle Measurement

Silicone (SilfloR, Flexico, Colchester, UK) was used for to make dorsal skin replicas of the hairless mice. The thin silicone gel print of skin surfaces were confirmed by using the visiometer technique. To recording of images, we used a charge-coupled device camera and analyzed using Skin Visioline VL650 software (Courage and Khazaka, Cologne, Germany). The data used to evaluate skin wrinkles were the average depth and length.

### 4.10. Statistical Analysis

All measurements were repeated three times. Results are expressed as means ± standard deviation and statistically analyzed by ANOVA and Duncan’s multiple range tests. Statistical significance was set at *p* < 0.05.

## 5. Conclusions

In this study, our data provides evidence indicating that UMH extracts attenuated H_2_O_2_ and UVB-induced skin photo-aging by activating antioxidant enzymes and inhibiting MAPK pathways. According to our present results, UMH is expected to take advantage of the functional material in antioxidant and the prevention of skin damage caused by ROS.

## Abbreviations

ROS	Reactive oxygen species
SOD	Susawperoxide dismutase
CAT	Catalase
GPX	Glutathione peroxidase
GR	Glutathione reductase
